# Supplementary material for: Tead1a Initiates Transcriptional Priming Through the TEAD1a/YAP‐Notch1‐Spi1/Cebpα Axis to Promote Neutrophil Fate
Source: Adv Sci (Weinh). 2025 Aug 28;12(41):e05441. doi: 10.1002/advs.202505441 (PMC12591127; doi:10.1002/advs.202505441)
Supplement: Supplementary file 4 — Supplementary Dataset [file ADVS-12-e05441-s004.zip › 3-7.zip!/3-7/02-7.cluster/cluster_heatmap_top9.pdf]

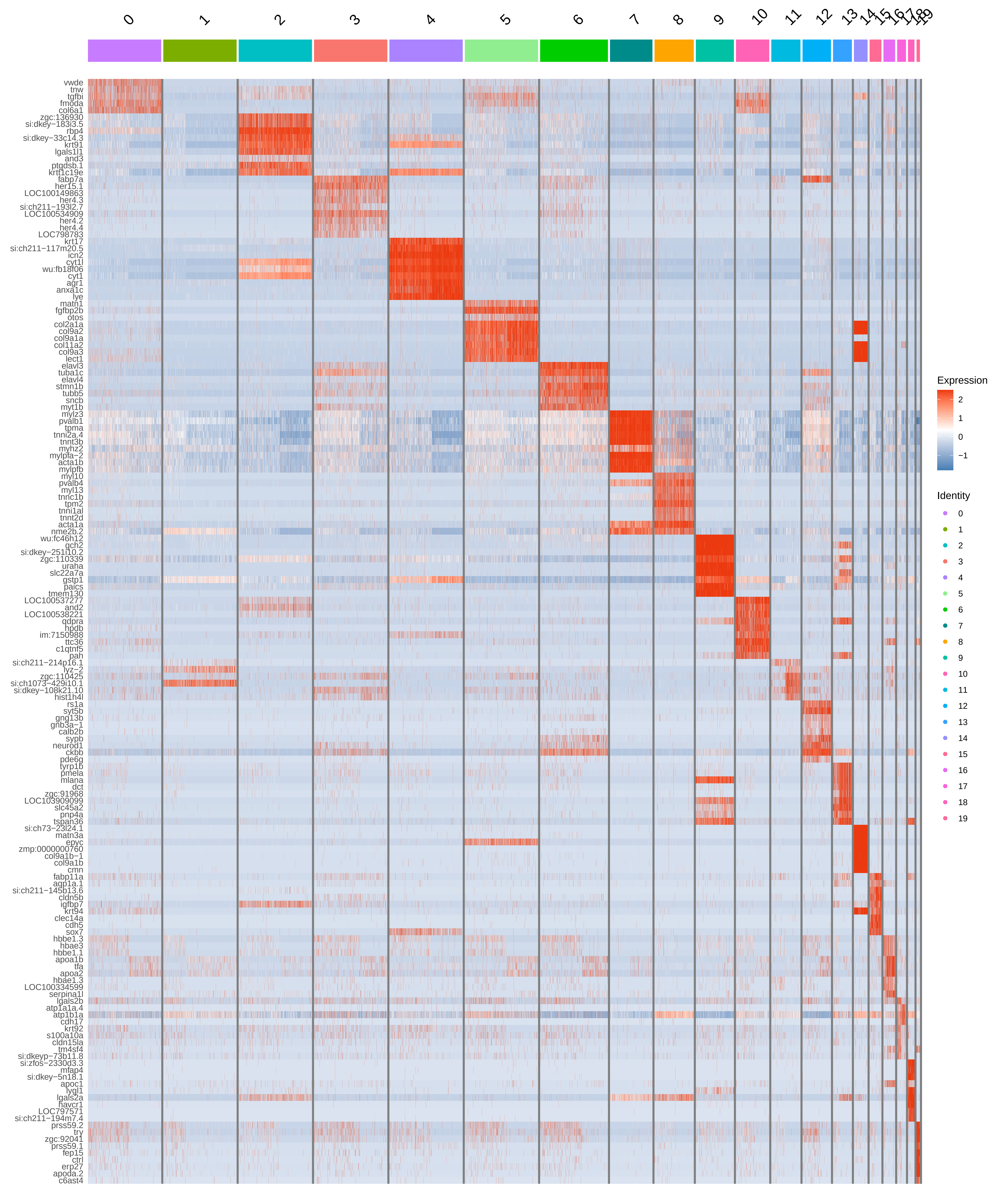
0
1
2
3
4
5
6
7
8
9
10
11
12
13
14
15
16
17
18
19
vwde
tnw
tgfbi
fmoda
col6a1
zgc:136930
si:dkey−183i3.5
rbp4
si:dkey−33c14.3
krt91
lgals1l1
and3
ptgdsb.1
krt1c19e
fabp7a
her15.1
LOC100149863
her4.3
si:ch211−193l2.7
LOC100534909
her4.2
her4.4
LOC798783
krt17
si:ch211−117m20.5
icn2
cyt1l
wu:fb18f06
cyt1
agr1
anxa1c
lye
matn1
fgfbp2b
otos
col2a1a
col9a2
col9a1a
col11a2
col9a3
lect1
elavl3
tuba1c
elavl4
stmn1b
tubb5
sncb
myt1b
mylz3
pvalb1
tpma
tnni2a.4
tnnt3b
myhz2
mylpfa−2
acta1b
mylpfb
myl10
pvalb4
myl13
tnnc1b
tpm2
tnni1al
tnnt2d
acta1a
nme2b.2
wu:fc46h12
gch2
si:dkey−251i10.2
zgc:110339
uraha
slc22a7a
gstp1
paics
tmem130
LOC100537277
and2
LOC100538221
qdpra
hpdb
im:7150988
ttc36
c1qtnf5
pah
si:ch211−214p16.1
lyz−2
zgc:110425
si:ch1073−429i10.1
si:dkey−108k21.10
hist1h4l
rs1a
syt5b
gng13b
gnb3a−1
calb2b
sypb
neurod1
ckbb
pde6g
tyrp1b
pmela
mlana
dct
zgc:91968
LOC103909099
slc45a2
pnp4a
tspan36
si:ch73−23l24.1
matn3a
epyc
zmp:0000000760
col9a1b−1
col9a1b
cmn
fabp11a
agp1a.1
si:ch211−145b13.6
cldn5b
igfbp7
krt94
clec14a
cdh5
sox7
hbbe1.3
hbae3
hbbe1.1
apoa1b
tfa
apoa2
hbae1.3
LOC100334599
serpina1l
lgals2b
atp1a1a.4
atp1b1a
cdh17
krt92
s100a10a
cldn15la
tm4sf4
si:dkeyp−73b11.8
si:zfos−2330d3.3
mfap4
si:dkey−5n18.1
apoc1
lygl1
lgals2a
havcr1
LOC797571
si:ch211−194m7.4
prss59.2
try
zgc:92041
prss59.1
fep15
ctrl
erp27
apoda.2
c6ast4
Expression
2
1
0
−1
Identity
0
1
2
3
4
5
6
7
8
9
10
11
12
13
14
15
16
17
18
19
